# Dietary stilbenes as modulators of specific miRNAs in prostate cancer

**DOI:** 10.3389/fphar.2022.970280

**Published:** 2022-08-24

**Authors:** Anait S. Levenson

**Affiliations:** College of Veterinary Medicine, Long Island University, Brookville, NY, United States

**Keywords:** stilbenes, miRNAs, MTA1, chemoprevention, interception, biomarkers, active surveillance, prostate cancer

## Abstract

Accumulated experimental data have suggested that natural plant products may be effective miRNA-modulating chemopreventive and therapeutic agents. Dietary polyphenols such as flavonoids, stilbenes, and lignans, among others, have been intensively studied for their miRNA-mediated cardioprotective, antioxidant, anti-inflammatory and anticancer properties. The aim of this review is to outline known stilbene-regulated miRNAs in cancer, with a special focus on the interplay between various miRNAs and MTA1 signaling in prostate cancer. MTA1 is an epigenetic reader and an oncogenic transcription factor that is overexpressed in advanced prostate cancer and metastasis. Not surprisingly, miRNAs that are linked to MTA1 affect cancer progression and the metastatic potential of cells. Studies led to the identification of MTA1-associated pro-oncogenic miRNAs, which are regulated by stilbenes such as resveratrol and pterostilbene. Specifically, it has been shown that inhibition of the activity of the MTA1 regulated oncogenic miR-17 family of miRNAs, miR-22, and miR-34a by stilbenes leads to inhibition of prostatic hyperplasia and tumor progression in mice and reduction of proliferation, survival and invasion of prostate cancer cells *in vitro*. Taken together, these findings implicate the use of resveratrol and its analogs as an attractive miRNA-mediated chemopreventive and therapeutic strategy in prostate cancer and the use of circulating miRNAs as potential predictive biomarkers for clinical development.

## Introduction

Countless efforts are aimed towards the development of cancer chemopreventive and therapeutic strategies with the use of natural bioactive polyphenols such as a large group of flavonoids, stilbenes, lignans, phenolic acids, and others (Asensi et al., 2011; Kang N.J et al., 2011; Miyata et al., 2019; Forni et al., 2021; Rudrapal et al., 2022). Polyphenols act on multiple targets in signaling pathways related to inflammation, oxidative stress and DNA damage, carcinogenesis, tumor cell proliferation, angiogenesis, and metastasis (Asensi et al., 2011; Zhao et al., 2020; Khan et al., 2021; Rudrapal et al., 2022). Studies over the past two decades have led to the development of the concept of “epigenetic prevention and therapy” for cancer, which includes regulation of genes by non-coding RNAs (ncRNAs) (Shukla et al., 2019; Levenson, 2021). Non-coding RNAs regulate chromatin architecture (Taft et al., 2009) and control gene expression by negatively regulating gene expression through a number of mechanisms (Filipowicz et al., 2008). Among ncRNA, microRNAs (miRNAs, miRs) are small, single-stranded, sequence-specific RNAs that induce degradation or inhibit translation of their target mRNAs (Ventura and Jacks, 2009). MiRNAs modulations have been shown to be involved in many pathologies including inflammation and cancer. In cancer, miRNAs are designated as oncogenic (oncomiRs), which downregulate tumor suppressor (TS) or other genes involved in cell differentiation thereby contributing to tumor formation, or oncosuppressor miRs, which downregulate proteins with oncogenic activity (Shenouda and Alahari, 2009). MiRNAs expressed in a tissue-specific manner have a multi-target mode of action (Link et al., 2010). MiRNAs are gaining increasing attention as potential noninvasive diagnostic, prognostic, and predictive biomarkers in cancer because they are stable, highly sensitive, and easily detectable in extracellular fluids (Quirico and Orso, 2020; Wang J et al., 2020).

Numerous studies have investigated miRNA profiles and their regulation by dietary phytochemicals, and the accumulated data implicate natural polyphenols as attractive miRNA-mediated chemopreventive and therapeutic strategy options in solid tumors and hematological cancers (Dhar et al., 2011; Vanden Berghe, 2012; Kumar et al., 2016; Levenson and Kumar, 2020; Levenson, 2021). Prostate cancer is ideal disease for nutritional chemoprevention in the general population of elderly men due to its slow progression in and dependence on diet (Peisch et al., 2017; Matsushita et al., 2020). Unfortunately, the incidence of low-risk prostate cancer has increased in the last two decades. Active surveillance is accepted as a management option for favorable-risk prostate cancer. However, due to the heterogeneity of this population, the lack of personalized risk assessments and the absence of treatment, the long-term outcome of active surveillance is not satisfying for approximately 30% of patients (Dhawan et al., 2016; Moschini et al., 2017; Overland et al., 2019; Pastor-Navarro et al., 2021). Since active surveillance patients are diagnosed with either prostatic intraepithelial neoplasia (PIN) or early-stage cancer (Gleason <6), it is apparent that various carcinogenic signaling pathways are already activated and can lead to pathological or clinical progression. Thus, the active surveillance subpopulation of patients with varying cancer risks may benefit from more active, clinical-based nutritional chemopreventive, i.e, interceptive strategies. Natural bioactive compounds known for their anti-inflammatory, antioxidant and anticancer effects may represent risk-reducing agents recommended for cancer interception (Blackburn, 2011). Nutritional interception in intermediate- and high-risk active surveillance patients should target specific molecular pathways. Particularly, our group has shown that natural dietary stilbenes exhibit targeted chemoprotective, interceptive and therapeutic effects against prostate cancer *in vitro* and *in vivo* (Kai et al., 2010; Li et al., 2013; Dhar et al., 2016; Butt et al., 2017; Kumar et al., 2019; Gadkari et al., 2020; Levenson, 2020; Hemani et al., 2022). This review will focus on the potential of natural stilbenes to protect against prostate cancer through the modulation of specific miRNAs that can conceivably be detected in bloodstream and urine and serve as prognostic and predictive biomarkers for certain populations of prostate cancer patients in the future.

### Stilbenes, miRNAs and cancer

#### Dietary stilbenes

**FIGURE 1:**
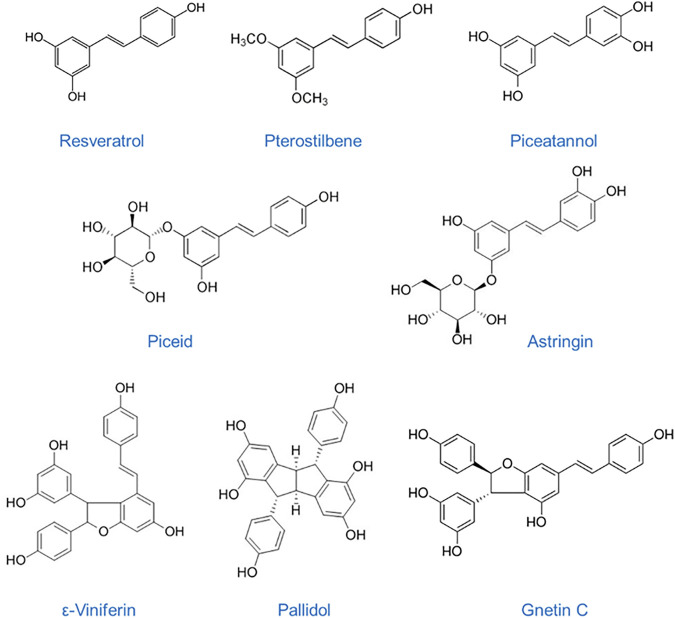
Chemical structures of dietary stilbenes: Resveratrol, *trans*-3, 4′, 5-trihydroxystilbene, MW: 228.24 g/mol; Pterostilbene, *trans*-3,5-dimethoxy-4′-hydroxystilbene, MW: 256.30 g/mol; Piceatannol, *trans*-3,5,3′4′-tetrahydroxystilbene, MW: 244.24 g/mol; Piceid, *trans*-resveratrol-3-*O*-glucoside, MW: 390.39 g/mol; Astringin, *trans*-piceatannol-3-β-D-glucuronide, MW: 406.38 g/mol; ε-Viniferin, *trans*-epsilon-resveratrol dimer, MW: 454.50 g/mol; Pallidol, tetracyclic homodimer resveratrol, MW: 454.48 g/mol; Gnetin C, dimer resveratrol, MW: 454.48 g/mol.

Stilbenes are a class of polyphenols naturally found in a wide variety of a small and heterogeneous group of plants, including *Vitis vinifera*, *Vaccinium, Cissus adnata*, *Fallopia japonica*, *Polygonum* *cuspidatum*, and *Picea sitchensis* and *abies*, to mention some (Rimando et al., 2004; Riviere et al., 2012). More than 400 natural stilbenoids have been identified in plants (Shen et al., 2009) where they are produced in response to biotic and abiotic environmental stresses (Bavaresco, 2003). Family members of the stilbenoids have a C6-C2-C6 basic skeleton and consist of two or more phenolic rings linked by an ethane double bond. Natural stilbenes are composed of resveratrol derivatives and concentrated in dietary sources such as grapes, red wine, grape juice, peanuts, berries, passion fruit and some medicinal plants. There are several well-known dietary stilbenes such as resveratrol, piseid, resveratrol dimers pallidol, viniferins, and Gnetin C, pterostilbene, piceatannol, and astringin (Figure 1). Resveratrol (C_14_H_12_O_3_) is the most widely studied stilbene compound, found mostly in grapes and red wine but also in cranberries, pistachios, and chocolate, has multiple and diverse pharmacological properties including antioxidant, anti-inflammatory and anticancer properties (Rauf et al., 2018; Galiniak et al., 2019; Kataria and Khatkar, 2019; Rudrapal, 2022). Pterostilbene (C_16_H_16_O_3_) is a naturally occurring methoxylated analog of resveratrol, found in blueberries and grapes, with improved pharmacokinetic efficacy and more potent biological efficacy over resveratrol (Rimando et al., 2002; Rimando et al., 2004; Rimando et al., 2005; Kapetanovic et al., 2011; Hagiwara et al., 2012; Dhar et al., 2015b). Piceatannol (C_14_H_12_O_4_) is another naturally occurring resveratrol metabolite, found in red wine, grapes, white tea or passion fruit, s recognized as a strong tyrosine kinase inhibitor in different types of cancer (Su and David, 2000; Vo et al., 2010; Kang C.H et al., 2011). Piceid, a major resveratrol glucoside in grape juices, demonstrated antiproliferative effects in epithelial and cancer cells (Su et al., 2013; Storniolo et al., 2014; Zhang T. et al., 2019). Piceatannol’s glucoside, astringin (C_20_H_22_O_9_), especially found in red wine, has been shown to function as potential cancer-chemopreventive agent by a mechanism different from that of resveratrol (Waffo-Teguo et al., 2001). Moreover, a recent report demonstrated potent anti-angiogenic effect of astringin as well as of resveratrol dimers ε-viniferin, ω-viniferin, and pallidol (C_28_H_22_O_6_), also concentrated in grapes and red wine (Fernandez-Cruz et al., 2019). Gnetin C (C_28_H_22_O_6_), a resveratrol dimer found in melinjo (*Gnetum gnemon*) seed extracts that are commonly used in Indonesian cuisine, possesses a broad spectrum of the same pharmacological activities as other stilbenes with the advantage of superior bioavailability and biological efficacy (Narayanan et al., 2015; Kumar et al., 2019; Gadkari et al., 2020). Chemopreventive effects of dietary stilbenes are mainly due to their anti-inflammatory and antioxidant activity but also direct targeting of multiple signaling pathways involved in carcinogenesis, cancer progression and metastasis. Importantly, accumulated data suggest that the beneficial effects of dietary stilbenes may be in part attributed to their epigenetic properties, including regulation of DNA methylation, histone modifications, and, notably, modulation of the expression of miRNAs/miRs (Kumar et al., 2015; Kumar et al., 2016; Kumar and Levenson, 2018; Levenson, 2021).

### Stilbene-regulated miRNAs in cancer

**FIGURE 2:**
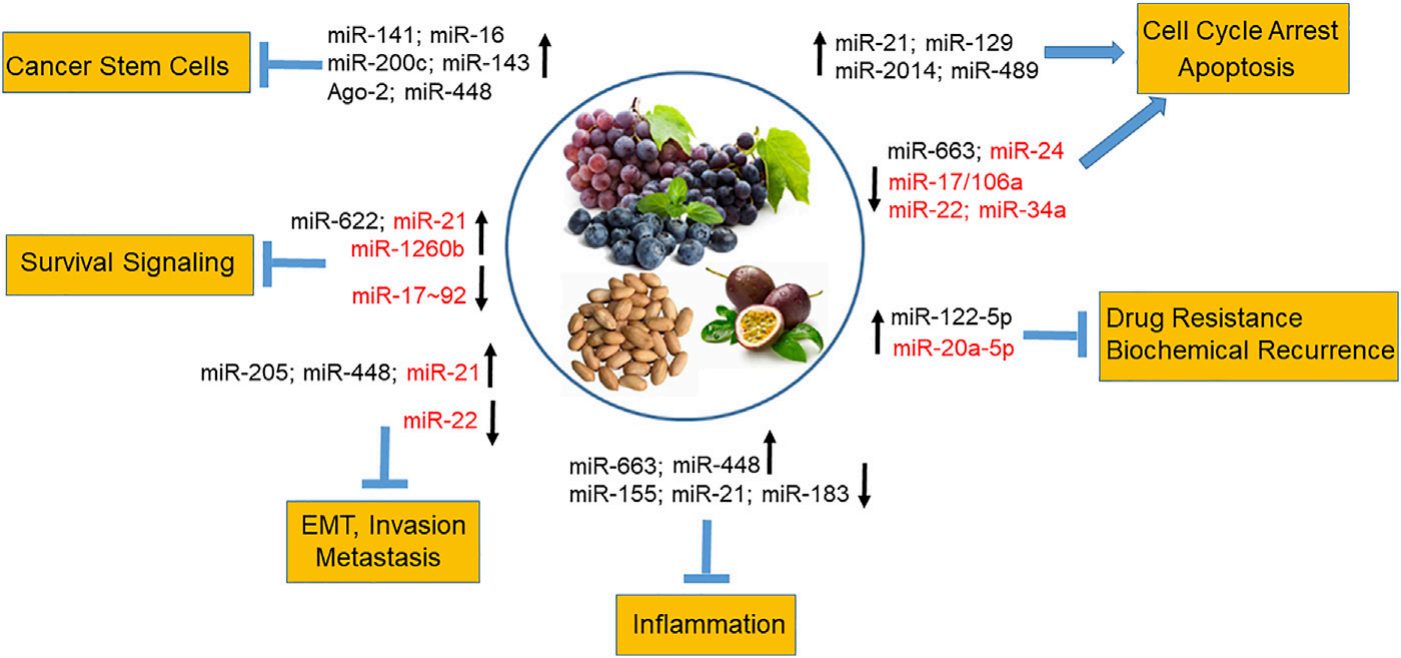
MiR-mediated biological effects of dietary stilbenes in cancer. Stilbenes from dietary sources such as grapes, blueberries, peanuts, and passion fruits modulate expression of miRNAs involved in promoting cell cycle arrest and apoptosis and inhibiting inflammation, survival pathways, cancer drug resistance and recurrence, invasion and metastasis as well as reducing cancer stem cell population. The stilbene-regulated miRNAs in prostate cancer are shown in red.

In a high-throughput analysis of miRNA expression in response to stilbenes, modulated miRs were identified as essential for controlling cancer growth, survival, metabolism, motility, apoptosis, angiogenesis, and metastasis (Tili and Michaille, 2011). In general, studies have shown that stilbenes inhibit oncomiRs and upregulate oncosuppressor miRs, For example, resveratrol has been shown to upregulate miR-21, miR-129, miR-2014, and miR-489 in rodent mammary tumors tissues (Qin et al., 2014) and a number of other oncosuppressor miRs, causing cell cycle arrest and cell death in breast cancer cells (Venkatadri et al., 2016). In chemoresistant breast cancer cells, resveratrol restored cell chemosensitivity by upregulating miR-122-5p (Zhang W. et al., 2019). Resveratrol and pterostilbene reduced the cancer stem-like cells population in mammary tumor formation *in vivo* by upregulating the expression of oncosuppressor miR-141, miR-16, miR-200c, miR-143 and Ago-2, a key regulator of miRNA homeostasis and biogenesis (Hagiwara et al., 2012). Seventy-one miRNAs were regulated in human lung cancer cells by resveratrol (Bae et al., 2011) including TS miR-622, which targets K-ras (Han et al., 2012). The miRNA-mediated link between inflammation and cancer under resveratrol treatment was demonstrated by revealing upregulation of anti-inflammatory and oncosuppressor miRNAs, such as miR-663, and downregulation of pro-inflammatory and oncogenic miRs, such as miR-155 and miR-21 (Tili et al., 2010a; Tili et al., 2011; Latruffe et al., 2015). Importantly, studies have reported subsequent mechanistic experiments, in which the identification of mRNA target transcripts whose levels were modified by stilbenoids, were validated on mRNA and/or protein levels. For example, resveratrol anti-inflammatory activity occurred *via* targeting the miR-663-mediated AP-1 signaling pathway (Tili et al., 2010a) and through modulating miRs involved in TGFβ signaling in human colorectal cancer cells (Tili et al., 2010b). Pterostilbene inhibited tumor growth and metastasis in breast cancer xenografts *via* induction of miR-205 expression, which targeted the Src/Fak signaling (Su et al., 2015) and suppressed glioblastoma through the miR-205/GRP78 axis (Huynh et al., 2015). A different study found that pterostilbene–induced modulation of miR-448/NF-κB axis resulted in suppression of the generation of breast cancer stem cells and metastatic potential (Mak et al., 2013). Further, pterostilbene downregulation of oncogenic miR-663, whose expression is correlated with poor prognosis in endometrial cancer patients, led to induction of pro-apoptotic BCL2L14 in endometrial cancer cells *in vitro* (Wang et al., 2017). Another natural analog of resveratrol, piceatannol, exhibited inhibition of colorectal cancer cell growth and induction of apoptosis by inducing miR-129-mediated downregulation of BCL-2 (Zhang et al., 2014). Furthermore, piceatannol inhibited Sp-1-mediated ADAM17 expression and the TNFα/NF-kB pathway in human leukemia cells by downregulating Akt/Foxo3-mediated miR-183 expression (Liu and Chang, 2012). Figure 2 summarizes reported miR-mediated biological effects of stilbenes in various cancers including prostate cancer (see below).

### Stilbene-regulated miRNAs in prostate cancer

In prostate cancer, oncomiRs expression correlates with high Gleason score and clinical recurrence (Ambs et al., 2008; Brase et al., 2011) and can be easily detected in serum, plasma and urine (Ambs et al., 2008; Selth et al., 2012; Sapre and Selth, 2013; Jeon et al., 2020; Hasanoglu et al., 2021), which signifies future utilization of miR-regulated pathways as potential targets as well as prognostic and predictive biomarkers.

Differential miRNA expression profiling in LNCaP prostate cancer cells treated with resveratrol revealed considerable modulation of a set of 51 miRNAs, from which 23 miRNAs (putative oncomiRs) were significantly downregulated and 28 miRNAs (putative oncosuppressor miRs) were significantly upregulated (Dhar et al., 2011).

The downregulated by resveratrol miRs included miR-7, miR-24, miR-1260 and miR-17∼92 (miR-17, miR-20a, miR-20b, miR-92b) and miR-106ab clusters. Subsequently, a growing body of evidence has revealed that many of these miRNAs act as oncogenes in prostate cancer. For instance, miR-7 has been found to be upregulated in castrate-resistant prostate cancer (CRPC) clinical samples (Xie and Jiang, 2015). Downregulation of miR-24 induced apoptosis in DU145 prostate cancer cells by upregulating its target pro-apoptotic FAF1 protein (Qin et al., 2010). Multiple miRNAs-TGFβ checkpoints that control TGFβ/SMAD signaling in progression of prostate cancer were identified (Javed et al., 2020). For instance, overexpression of oncomiR-1260b resulted in reduced levels of tumor suppressor SMAD4 leading to prostate cancer progression. Interestingly, miR-1260b was also significantly downregulated by another natural polyphenol known for its anticancer properties, i.e. genistein that promoted SMAD4-mediated apoptosis in prostate cancer cells (Hirata et al., 2014). Overexpression of miR-20a has been detected in tumor tissue samples of prostate cancer patients with a Gleason score of 7–10 (Pesta et al., 2010). Moreover, a high miR-20a-5p expression in prostate tumor tissues was identified as one of the five miRNAs that may, as a panel, be used as a potential diagnostic biomarker (Damodaran et al., 2021) and as an independent predictor for biochemical recurrence (Stoen et al., 2021). The oncogenic role of miR-20a/CX43 (Li et al., 2012) and miR-20a/miR-17/ATG7 (Guo et al., 2016) in prostate cancer was also reported. The miR-17∼92 and paralogs miR-106a∼363 and miR-106b∼25 are commonly described as oncogenic in many cancers (Kolenda et al., 2020) including prostate cancer, in which they have been validated clinically as significantly upregulated compared to normal samples (Taylor et al., 2010). Importantly, miR-17∼92 and miR-106ab have been directly linked to the tumor suppressor PTEN (Olive et al., 2010; Poliseno et al., 2010), one of the frequently defective genes in primary and metastatic prostate cancer (Li et al., 1997). In consequent studies, using functional luciferase reporter assays, it was demonstrated that ectopically expressed miR-17, miR-20a and miR-106b directly target *Pten* 3′UTR to reduce its expression in DU145 and 22Rv1 prostate cancer cells. Notably, these effects were rescued upon treatment with resveratrol and pterostilbene (Dhar et al., 2015b). Moreover, pterostilbene treatment diminished the miR-17/106a-promoted tumor growth in DU145-Luc prostate cancer xenografts through miR-mediated upregulation of PTEN mRNA and protein levels in tumor tissues, causing apoptosis (Dhar et al., 2015b). A different report demonstrated resveratrol-induced reduction of prostate cancer growth and metastasis through Akt/miR-21/PDCD4 pathway (Sheth et al., 2012).

The upregulated by resveratrol miRs in prostate cancer included miR-1469, miR-612, miR-149, miR-638, miR-654-5p, miR-1908, miR-1915, miR-1231, miR-939, miR-671-5p (Dhar et al., 2011), many of which are currently documented as oncosuppressor miRs in several types of cancer (Jin et al., 2020; Li et al., 2021; Liu et al., 2020a, b; Tan et al., 2016). For example, a recent study demonstrated that miR-149 could inhibit AR expression and reduce the activity of PI3K/Akt1 signaling in castrate-resistant cells (Zhao et al., 2021) and also regulate RGS17-mediated oncogenic effects (Ma et al., 2021) revealing its tumor suppressor nature in prostate cancer. The tumor suppressive role of miR-1231 targeting EGFR in prostate cancer and reducing cell proliferation, migration, and invasion was recently demonstrated (Wang Y et al., 2020). The authors propose diminished miR-1231 levels as a prognostic biomarker for advanced prostate cancer. MiR-654-5p was among fifteen AR downregulating miRNAs that decreased androgen-induced proliferation of prostate cancer cells (Ostling et al., 2011). Another miR, upregulated by resveratrol (Dhar et al., 2011), namely miR-939-3p, was decreased in prostate cancer tissues and cell lines compared to normal adjacent tissues and normal epithelial cell line. MiR-939-3p acted as an oncosuppressor miR through the long non-coding RNA (lncRNA) brain cytoplasmic RNA1 BCYRN1/HDAC11 oncogenic axis in prostate cancer (Huo et al., 2020). Interesting results were reported on the overexpression of spermidine synthase (SRM) in prostate cancer tissues and miR-1908-mediated regulation of SRM, which controls the secretion of extracellular vesicles (EV) in prostate cancer (Urabe et al., 2020). Finally, upregulation of EV-associated miR-1915-3p was concomitant with improved survival time along with two other miRs, but only miR-1915-3p was associated with longer recurrence-free survival as an independent prognostic marker in prostate cancer patients with low and high Gleason scores and of various races (Ali et al., 2021). It is important to notice that detailed experimental mechanistic studies to prove biological consequences of stilbene modulated oncosuppressive miRs in prostate cancer are lucking. Of note, miR-1469 have been reported to be induced by genistein resulting in promotion of apoptosis *via* inhibition of the Mcl-1 pathway in laryngeal cancer cells (Ma et al., 2018).

The role of some miRs in cancer is complex and ambiguous: these miRs have been reported as both oncogenic and oncosuppressors. However, while this controversy can be attributed to the cell specificity, organ tissue microenvironment and various targets for a given miR, a disagreement about the behavior of a given miR in the same type of cancer is less understandable. For example, although recognized as a tumor suppressor in some tumors (Li et al., 2019; Xin et al., 2019; Wang et al., 2021) the unexpected oncogenic role of miR-671-5p associated with targeting tumor suppressor SOX6 in prostate cancer has been recently reported (Yu et al., 2018). Moreover, oncosuppressor miR-149 was found to be overexpressed in CRPC samples (Zhu et al., 2015) and has been seen as an oncogenic miR associated with syndecan-1 and inhibiting SOX2, NANOG, and Oct4 tumor suppressors (Fujii et al., 2015). Tables 1, 2 summarize confirmed oncogenic (Table 1) and oncosuppressor miRs (Table 2) in prostate cancer earlier identified as resveratrol-regulated (Dhar et al., 2011). The data regarding the functions and exact roles of identified stilbene-regulated miRNAs in prostate cancer are incomplete and require further studies.

**TABLE 1:** Confirmed Oncogenic miRNAs in Prostate Cancer^a^.

Stilbene	miRNA	Identified Target	Associated information or Event	References
No treatment	miR-7		Upregulated in CRPC clinical samples	Xie and Jiang, (2015)
	miR-24	FAF1	Apoptosis in DU145 cells	Qin et al. (2010)
“Genistein”	miR-1260b	SMAD4	Apoptosis in PC3 cells	Hirata et al. (2014)
No treatment	miR-20		Overexpressed in aggressive prostate cancer	Pesta et al. (2010)
			Potential diagnostic marker	Damodaran et al. (2021)
			Predictor for biochemical recurrence	Stoen et al. (2021)
No treatment	miR- 20	CX43	PCa-2b cell proliferation, tumor growth	Li. et al. (2012)
No treatment	AR/miR-20a/miR-17	ATG7	Autophagy	Guo et al. (2016)
No treatment	miR-17∼92		Upregulated in clinical samples	Taylor et al. (2010)
Resveratrol	miR-17∼92	PTEN	Reduced cell proliferation and xenograft tumor growth	Dhar et al. (2015b)
Pterostilbene				
Resveratrol	miR-106a∼363	PTEN	Reduced cell proliferation and xenograft tumor growth	Dhar et al. (2015b)
Pterostilbene				
Resveratrol	miR-106b∼25	PTEN	Reduced cell proliferation and xenograft tumor growth	Dhar et al. (2015b)
Pterostilbene				
Resveratrol	Akt/miR-21	PDCD4	Cancer growth and metastasis	Sheth et al. (2012)
Grape extract-Diet	MTA1/c-miR-34a	p53	Reduced PIN in *Pten* ^*+/f*^ *; Cre* ^*+*^ mice	Joshi et al. (2020)
	MTA1/c-miR-22	p21		
Pterostilbene-Diet	MTA1/c-miR-34a		Reduced hgPIN in *R26* ^*MTA1*^ *; Pten* ^*+/f*^ *; Cre* ^*+*^ mice	Hemani et al. (2022)
	MTA1/c-miR-22			
No treatment	MTA1/miR-22	E-cadherin	Invasion in RWPE1 & LNCaP cells	Dhar et al. (2017)

a These miRNAs, are putative stilbene-regulated onco-miRs previously identified by Dhar et al. (2011); c-miR, circulating miR detected in serum.

**TABLE 2:** Confirmed Oncosuppressor miRNAs in Prostate Cancer^a^.

Stilbene	miRNA	Identified Target	Associated information or Events	References
No treatment	miR-654-5p	AR	PSA, LNCaP cell proliferation	Ostling et al. (2011)
No treatment	miR-149	AR	CRCP 22-Rv1 cells	Zhao et al. (2021)
		P13K/Akt1		
No treatment	miR-149-5p	RGS17	Viability, proliferation, migration of 22Rv1 and C4-2 cells , high in prostate cancer tissues	Ma et al. (2021)
No treatment	miR-1231	EGFR	Cell proliferation, migration, invasion in DU145, 22Rv1, PC3, and VCaP, high in prostate cancer tissues	Wang Y et al. (2020)
No treatment	miR-939-3p	BCYRN1/HDAC11	Cell proliferation,	Huo et al. (2020)
			Decreased in prostate cancer tissues vs. normal	
No treatment	miR-1908	SRM	22Rv1 cells, high in prostate cancer tissues	Urabe et al. (2020)
No treatment	miR-1915-3p		Upregulation was linked to recurrence-free survival	Ali et al. (2021)
			Independent prognostic marker	

a These miRNAs are putative stilbene-regulated oncosuppressor miRs previously identified by Dhar et al., 2011.

### Stilbenes as inhibitors of MTA1 signaling and associated miRNAs in prostate cancer

Understanding the specific molecular mechanisms of premalignancy and cancer progression opens opportunities for developing targeted interceptive measures. It is largely acknowledged that natural polyphenols, particularly stilbenes have pleiotropic anti-inflammatory and anticancer effects acting through various signal transduction pathways such as NF-kB, AP-1, and MAPK signaling among others.

**FIGURE 3:**
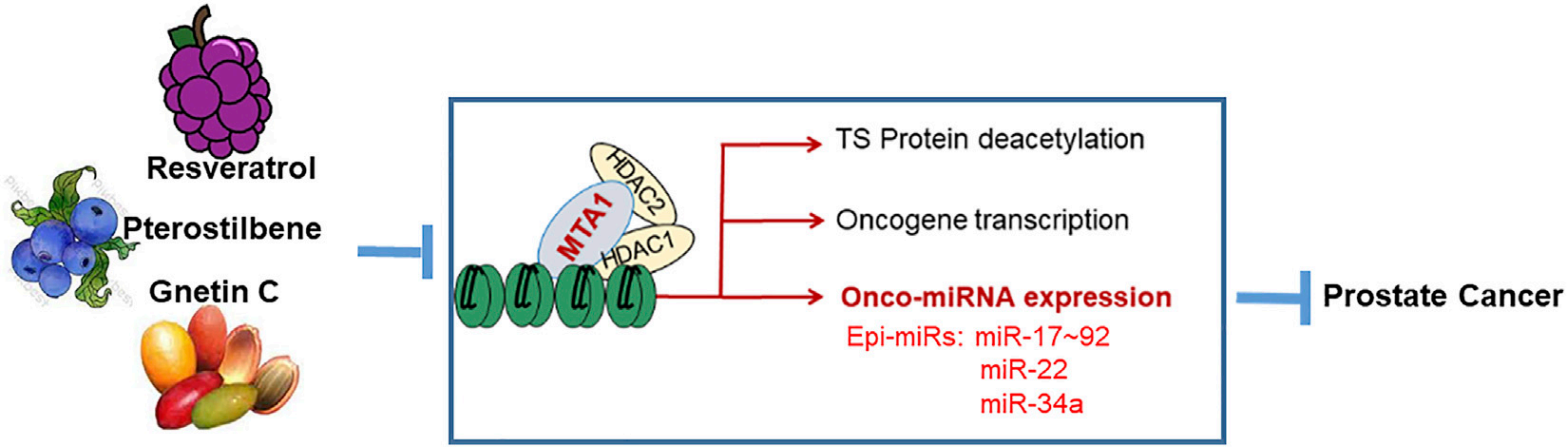
Effects of dietary stilbenes on the MTA1 signaling pathways including miRNAs. The MTA1/HDAC1,2 signaling is triggered by prostate cancer progression and involves deacetylation of some tumor suppressor (TS) proteins (p53 and PTEN), transcription of oncogenes, and activation of oncogenic miRNAs. These cascades can be inhibited by the dietary stilbenes accumulated in grapes, blueberries and melinjo berries.

In prostate cancer, metastasis associated protein 1 (MTA1) signaling is aberrantly activated due to overexpression of MTA1 and activation of associated pathways. Increased expression of MTA1 is associated with high Gleason score, recurrence, and metastasis in prostate cancer (Hofer et al., 2004; Dias et al., 2013). MTA1 plays a critical role in different stages of prostate cancer, including chronic inflammation, tumor growth, epithelial-to-mesenchymal transition (EMT), invasion, migration, angiogenesis and metastasis (Levenson et al., 2014). Studies have demonstrated the MTA1-mediated chemopreventive and therapeutic effects of natural stilbenes in prostate cancer (Levenson, 2020). Both resveratrol and pterostilbene inhibited survival pathways and induced apoptosis in prostate cancer through downregulation of the MTA1/HDAC1, 2 units of the NuRD complex, which resulted in the promotion of acetylation and reactivation of tumor suppressors p53 and PTEN (Kai et al., 2010; Dhar et al., 2015b). The inhibitory effects of resveratrol and pterostilbene on MTA1-mediated angiogenesis involving HIF-1a, VEGF, and IL-1β were also consistently reported (Kai et al., 2011; Li et al., 2013; Dhar et al., 2016; Butt et al., 2017; Hemani et al., 2022). We have also shown the MTA1-mediated and MTA1 independent chemopreventive and therapeutic value of Gnetin C in prostate cancer *in vitro* and *in vivo* (Kumar et al., 2019; Gadkari et al., 2020). Importantly, stilbenes can inhibit MTA1-associated oncogenic miRNAs (Figure 3).

MTA1, an epigenetic reader and “master co-regulator” and transcription factor, plays a role in direct or indirect transcriptional activation/suppression of specific genes including miRNAs (Manavathi and Kumar, 2007). MiRNAs that are regulated by epigenetic factors are called Epi-miRs (Valeri et al., 2009; Dhar et al., 2017; Levenson, 2020). MTA1-associated stilbene-regulated Epi-miRNAs have been identified by a systematic analysis and review of the results from the following three high-throughput analyses in prostate cancer: the identification of resveratrol responsive miRNAs using miRNA microarrays in LNCaP cells (Dhar et al., 2011), the identification of MTA1-associated miRNAs by differential miRNA microarrays profiling in LNCaP MTA1 knockdown cells (Dhar et al., 2017), and the identification of prostate tissues transcriptional targets of MTA1 by ChIP-Seq from prostate-specific *Pten*-deficient mice fed with pterostilbene-diet (Dhar et al., 2016).

Our special interest in oncomiR/PTEN axis came from previous studies, in which we and others showed upregulation of PTEN protein levels by resveratrol in prostate cancer cells (Wang et al., 2010; Dhar et al., 2011), one mechanism of which was the inhibition of MTA1-mediated deacetylation and inactivation of PTEN (Dhar et al., 2015a). In parallel, resveratrol downregulated miR-17-92, miR-106a∼363, and miR-106b∼25 clusters in prostate cancer cells (Dhar et al., 2011). Further, miR-17, miR-20a, and miR-106b directly targeted the 3′UTR of Pten, an event that was reversed by resveratrol and pterostilbene in prostate cancer cells (Dhar et al., 2015b). Moreover, the reduced levels of circulating miR-17 and miR-106a in the sera from mice treated with pterostilbene - led to reduced tumor growth and revealed the potential of stilbene-responsive miRs as chemopreventive and predictive biomarkers in prostate cancer (Dhar et al., 2015b). In addition, miRNA profiling of MTA1-knockdown cells revealed direct regulation of miR-92b by MTA1, among others (Dhar et al., 2017).

Other candidate MTA1-associated stilbene-regulated miRs are miR-22 and miR-34a (Dhar et al., 2017). According to publicly available prediction algorithms, oncomiRs miR-22 and miR-34a target tumor suppressors p21 and p53, respectively. An inverse relationship between MTA1/miR-22 and p21 and MTA1/miR-34a and p53 was demonstrated in MTA1 knockdown prostate cancer cells (Joshi et al., 2020). Notably, MTA1-associated miR-22 and miR-34a were regulated by low-fat and high-fat diets supplemented with grape powder fed to mice prone to developing PIN (*Pten* ^*+/f*^ *, Pb-Cre* ^*+*^). These two PIN-derived circulating oncomiRs further were detected in murine serum, in which they showed statistically significant reduced levels in mice fed with diets supplemented with grape powder containing not only stilbenes but other polyphenols (Joshi et al., 2020). In a different set of experiments using a high-risk premalignant prostate cancer mouse model (*R26* ^*MTA1*^ *; Pten* ^*+/f*^ *; Pb-Cre* ^*+*^ *)* fed a diet supplemented with pterostilbene (100 mg/kg diet), we registered MTA1-targeted chemoprevention along with reduced circulating miR-22 and miR-34a levels in response to pterostilbene treatment (Hemani et al., 2022). As the same miR can target multiple mRNAs, studies have reported miR-22 direct targeting of PTEN in prostate cancer (Poliseno et al., 2010) and downregulation of PTEN protein levels in RWPE1 prostate cancer cells ectopically expressing miR-22 (Dhar et al., 2017). Another valuable MTA1-associated miRNA/target axis was identified from miRNA profiling studies and was functionally validated in prostate cancer: the miR-22/E-cadherin axis (Dhar et al., 2017). E-cadherin is a valid adhesion factor that plays an essential role against EMT leading to invasion and metastasis (Frixen et al., 1991; Perl et al., 1998; Onder et al., 2008). In addition to correlative observation of aggressive DU145 and PC3M prostate cancer cells with high MTA1/miR-22 and low E-cadherin expression compared to “good” RWPE1 and LNCaP cells, meta-analysis of patient tumor samples indicated a positive correlation between MTA1 and miR-22 and a negative correlation between MTA1 and E-cadherin, supporting their inhibitory effect on E-cadherin expression. MTA1-induced drastic downregulation of E-cadherin was further shown in the prostate tissues of prostate-specific transgenic mice overexpressing MTA1 (*R26* ^*MTA1*^ *; Pb-Cre* ^*+*^). Mechanistically, MTA1-induced overexpression of miR-22 reduced expression of E-cadherin resulting in increased cell invasiveness and migration of prostate cancer cells and the link between miR-22 and its putative target E-cadherin mRNA was demonstrated using reporter constructs of the 3ˊ-UTR of E-cadherin. MTA1-promoted miR-22-regulation of this adhesion factor makes the MTA1/miR-22/E-cadherin axis critical for promoting tumor invasiveness in prostate cancer cells (Dhar et al., 2017). Bearing in mind that miR-22 is a confirmed regulator of EMT (Song et al., 2013), its role as a prognostic and predictive biomarker in advanced prostate cancer and a potential therapeutic target becomes essential.

## Conclusion

Identifying miRNAs linked to specific signaling pathways that are critical for prostate tumor progression and metastasis might provide novel targeted chemoprevention and therapeutic opportunities. Pharmacological modulation of miRNA activities, specifically by dietary stilbenes may have tremendous impact in interceptive approaches for different stages of prostate cancer. The results from our studies suggest that miR regulation is conserved among stilbene family members and that the identified MTA1-miRNA network regulated by stilbenes plays a significant role in prostate cancer progression. These miRNAs are particularly attractive because they can be detected in serum or urine as “liquid biopsy” biomarkers essential for diagnosis, prediction of interception/therapy response, and prognosis in prostate cancer. Studies have also reported beneficial miR-mediated effects of stilbenes in combination with other natural polyphenols in various cancers. Due to their unique chemical structure, different classes of polyphenols may produce specific miR-mediated gene regulation, which may culminate in synergistic beneficial effects. Further studies have the potential of improving the goals of personalized medicine, specifically concerning personalized interception using miRNA-modulating natural products with potential chemopreventive and therapeutic benefits in prostate cancer.

## Glossary

ADAM17: disintegrin and metallopeptidase domain-containing protein 17
Akt: v-akt murine thymoma viral oncogene (protein kinase B)
AP1: jun proto-oncogene
AR: androgen Receptor
ATG7: autophagy related 7
Ago-2: argonaute 2
BCL-2: B-cell lymphoma 2
BCL2L14: B-cell lymphoma like 14
BCYRN1: brain cytoplasmic RNA 1
ChIP-Seq: chromatin immunoprecipitation sequencing
CRPC: castrate resistant prostate cancer
CX43: connexin 43 (Gap junction alpha 1 protein, GJA1)
DU145: human prostate cancer cell line
EGFR: epidermal growth factor receptor
EMT: epithelial-to-mesenchymal transition
Epi-miRNA: epigenetic miRNA (ribonucleic acid)
EV: extracellular vehicle
FAF1: fas-associated factor 1
Fak: focal adhesion kinase
Foxo3: forkhead box O3
GRP78: glucose-regulated protein 78
HDAC1/2: histone deacetylases 1 and 2
HDAC11: histone deacetylase 11
HIF-1α: hypoxia inducing factor 1
IL-1β: interleukin 1 Beta
K-ras: kirsten rat sarcoma virus
LNCaP: human prostate cancer cell line
lncRNA: long non-coding ribonucleic acid
Luc: luciferase
MAPK: mitogen-activated protein kinase
Mcl-1: myeloid cell leukemia 1
miR: microRNA (ribonucleic acid)
miRNA: microRNA (ribonucleic acid)
mRNA: messenger RNA (ribonucleic acid)
MTA1: metastasis-associated protein 1
mPIN: mouse PIN
NANOG: homeobox protein, a transcription factor in embryonic stem cells
ncRNA: non-coding RNA
NF-κB: nuclear factor kappa-light-chain-enhancer of activated B cells
NuRD: nucleosome remodeling and deacetylase complex
Oct4: homeobox protein, a transcription factor in embryonic stem cells
OncomiR: oncogenic miRNA
PC3: human prostate cancer cell line
PDCD4: programmed cell death 4
PI3K: phosphatidylinositol 3-kinase/serine/threonine kinase PKB
PIN: prostatic intraepithelial neoplasia
PTEN: phosphatase and tensin homolog deleted in chromosome 10
Ptenf/f: Pten gene, floxed
Pten+/f: Pten heterozygous mice (Pb-Cre4+, Pten+/f)
RGS17: regulator of G protein signaling
R26MTA1: rosa26 locus; R26MTA1: MTA1 overexpressing mice
22Rv1: human prostate cancer cell line
RWPE1: human prostate cancer cell line
SMAD4: mothers against decapentaplegic homolog 4
SOX2: SRY (sex determining region Y)-box 2
SOX6: SRY (sex determining region Y)-box 6
Sp-1: specificity protein 1
Src: proto-oncogene tyrosine-protein kinase
SRM: spermidine synthase
TGFβ1: transforming growth factor β1
TNFα: tumor necrosis factor alpha
TS: tumor suppressor
3′UTR: 3 prime untranslated region
VEGF: vascular endothelial growth factor

## References

[B1] Ali H. E. A. Gaballah M. S. A. Gaballa R. Mahgoub S. Hassan Z. A. Toraih E. A. (2021). Small extracellular vesicle-derived microRNAs stratify prostate cancer patients according to Gleason score, race and associate with survival of african American and caucasian men. Cancers (Basel) 13, 5236. 10.3390/cancers13205236 34680382 PMC8533757

[B2] Ambs S. Prueitt R. L. Yi M. Hudson R. S. Howe T. M. Petrocca F. (2008). Genomic profiling of microRNA and messenger RNA reveals deregulated microRNA expression in prostate cancer. Cancer Res. 68, 6162–6170. 10.1158/0008-5472.CAN-08-0144 18676839 PMC2597340

[B3] Asensi M. Ortega A. Mena S. Feddi F. Estrela J. M. (2011). Natural polyphenols in cancer therapy. Crit. Rev. Clin. Lab. Sci. 48, 197–216. 10.3109/10408363.2011.631268 22141580

[B4] Bae S. Lee E. M. Cha H. J. Kim K. Yoon Y. Lee H. (2011). Resveratrol alters microRNA expression profiles in A549 human non-small cell lung cancer cells. Mol. Cells 32, 243–249. 10.1007/s10059-011-1037-z 21887509 PMC3887628

[B5] Bavaresco L. (2003). Role of viticultural factors on stilbene concentrations of grapes and wine. Drugs Exp. Clin. Res. 29, 181–187. 15134373

[B6] Blackburn E. H. (2011). Cancer interception. Cancer Prev. Res. 4, 787–792. 10.1158/1940-6207.CAPR-11-0195 21636545

[B7] Brase J. C. Johannes M. Schlomm T. Falth M. Haese A. Steuber T. (2011). Circulating miRNAs are correlated with tumor progression in prostate cancer. Int. J. Cancer 128, 608–616. 10.1002/ijc.25376 20473869

[B8] Butt N. A. Kumar A. Dhar S. Rimando A. M. Akhtar I. Hancock J. C. (2017). Targeting MTA1/HIF-1α signaling by pterostilbene in combination with histone deacetylase inhibitor attenuates prostate cancer progression. Cancer Med. 6, 2673–2685. 10.1002/cam4.1209 29024573 PMC5673954

[B9] Damodaran M. Chinambedu D. M. SimonDuraiRaj, SandhyaSundaram, VenkatRamanan S. Ramachandran I. (2021). Differentially expressed miR-20, miR-21, miR-100, miR-125a and miR-146a as a potential biomarker for prostate cancer. Mol. Biol. Rep. 48, 3349–3356. 10.1007/s11033-021-06384-z 33948855

[B10] Dhar S. Hicks C. Levenson A. S. (2011). Resveratrol and prostate cancer: Promising role for microRNAs. Mol. Nutr. Food Res. 55, 1219–1229. 10.1002/mnfr.201100141 21714127

[B11] Dhar S. Kumar A. Gomez C. R. Akhtar I. Hancock J. C. Lage J. M. (2017). MTA1-activated Epi-microRNA-22 regulates E-cadherin and prostate cancer invasiveness. FEBS Lett. 591, 924–933. 10.1002/1873-3468.12603 28231399

[B12] Dhar S. Kumar A. Li K. Tzivion G. Levenson A. S. (2015a). Resveratrol regulates PTEN/Akt pathway through inhibition of MTA1/HDAC unit of the NuRD complex in prostate cancer. Biochim. Biophys. Acta 1853, 265–275. 10.1016/j.bbamcr.2014.11.004 25447541

[B13] Dhar S. Kumar A. Rimando A. M. Zhang X. Levenson A. S. (2015b). Resveratrol and pterostilbene epigenetically restore PTEN expression by targeting oncomiRs of the miR-17 family in prostate cancer. Oncotarget 6, 27214–27226. 10.18632/oncotarget.4877 26318586 PMC4694984

[B14] Dhar S. Kumar A. Zhang L. Rimando A. M. Lage J. M. Lewin J. R. (2016). Dietary pterostilbene is a novel MTA1-targeted chemopreventive and therapeutic agent in prostate cancer. Oncotarget 7, 18469–18484. 10.18632/oncotarget.7841 26943043 PMC4951302

[B15] Dhawan A. Graham T. A. Fletcher A. G. (2016). A computational modeling approach for deriving biomarkers to predict cancer risk in premalignant disease. Cancer Prev. Res. 9, 283–295. 10.1158/1940-6207.CAPR-15-0248 26851234

[B16] Dias S. J. Zhou X. Ivanovic M. Gailey M. P. Dhar S. Zhang L. (2013). Nuclear MTA1 overexpression is associated with aggressive prostate cancer, recurrence and metastasis in African Americans. Sci. Rep. 3, 2331–2341. 10.1038/srep02331 23900262 PMC3728596

[B17] Fernandez-Cruz E. Cerezo A. B. Cantos-Villar E. Richard T. Troncoso A. M. Garcia-Parrilla M. C. (2019). Inhibition of VEGFR-2 phosphorylation and effects on downstream signaling pathways in cultivated human endothelial cells by stilbenes from vitis spp. J. Agric. Food Chem. 67, 3909–3918. 10.1021/acs.jafc.9b00282 30892883

[B18] Filipowicz W. Bhattacharyya S. N. Sonenberg N. (2008). Mechanisms of post-transcriptional regulation by microRNAs: are the answers in sight? Nat. Rev. Genet. 9, 102–114. 10.1038/nrg2290 18197166

[B19] Forni C. Rossi M. Borromeo I. Feriotto G. Platamone G. Tabolacci C. (2021). Flavonoids: A myth or a reality for cancer therapy? Molecules 26, 3583. 10.3390/molecules26123583 34208196 PMC8230897

[B20] Frixen U. H. Behrens J. Sachs M. Eberle G. Voss B. Warda A. (1991). E-cadherin-mediated cell-cell adhesion prevents invasiveness of human carcinoma cells. J. Cell Biol. 113, 173–185. 10.1083/jcb.113.1.173 2007622 PMC2288921

[B21] Fujii T. Shimada K. Tatsumi Y. Fujimoto K. Konishi N. (2015). Syndecan-1 responsive microRNA-126 and 149 regulate cell proliferation in prostate cancer. Biochem. Biophys. Res. Commun. 456, 183–189. 10.1016/j.bbrc.2014.11.056 25462564

[B22] Gadkari K. Kolhatkar U. Hemani R. Campanelli G. Cai Q. Kumar A. (2020). Therapeutic potential of Gnetin C in prostate cancer: A pre-clinical study. Nutrients 12, 3631–3642. 10.3390/nu12123631 PMC7760540 33255879

[B23] Galiniak S. Aebisher D. Bartusik-Aebisher D. (2019). Health benefits of resveratrol administration. Acta Biochim. Pol. 66, 13–21. 10.18388/abp.2018_2749 30816367

[B24] Guo J. Mei Y. Li K. Huang X. Yang H. (2016). Downregulation of miR-17-92a cluster promotes autophagy induction in response to celastrol treatment in prostate cancer cells. Biochem. Biophys. Res. Commun. 478, 804–810. 10.1016/j.bbrc.2016.08.029 27501757

[B25] Hagiwara K. Kosaka N. Yoshioka Y. Takahashi R. U. Takeshita F. Ochiya T. (2012). Stilbene derivatives promote Ago2-dependent tumour-suppressive microRNA activity. Sci. Rep. 2, 314. 10.1038/srep00314 22423322 PMC3304512

[B26] Han Z. Yang Q. Liu B. Wu J. Li Y. Yang C. (2012). MicroRNA-622 functions as a tumor suppressor by targeting K-Ras and enhancing the anticarcinogenic effect of resveratrol. Carcinogenesis 33, 131–139. 10.1093/carcin/bgr226 22016468

[B27] Hasanoglu S. Goncu B. Yucesan E. Atasoy S. Kayali Y. Ozten Kandas N. (2021). Investigating differential miRNA expression profiling using serum and urine specimens for detecting potential biomarkers for early prostate cancer diagnosis. Turk. J. Med. Sci. 51, 1764–1774. 10.3906/sag-2010-183 33550766 PMC8569761

[B28] Hemani R. Patel I. Inamdar N. Campanelli G. Donovan V. Kumar A. (2022). Dietary pterostilbene for MTA1-targeted interception in high-risk premalignant prostate cancer. Cancer Prev. Res. 15, 87–100. 10.1158/1940-6207.CAPR-21-0242 PMC8828670 34675064

[B29] Hirata H. Hinoda Y. Shahryari V. Deng G. Tanaka Y. Tabatabai Z. L. (2014). Genistein downregulates onco-miR-1260b and upregulates sFRP1 and Smad4 *via* demethylation and histone modification in prostate cancer cells. Br. J. Cancer 110, 1645–1654. 10.1038/bjc.2014.48 24504368 PMC3960620

[B30] Hofer M. D. Kuefer R. Varambally S. Li H. Ma J. Shapiro G. I. (2004). The role of metastasis-associated protein 1 in prostate cancer progression. Cancer Res. 64, 825–829. 10.1158/0008-5472.can-03-2755 14871807

[B31] Huo W. Qi F. Wang K. (2020). Long noncoding RNA BCYRN1 promotes prostate cancer progression *via* elevation of HDAC11. Oncol. Rep. 44, 1233–1245. 10.3892/or.2020.7680 32705287

[B32] Huynh T. T. Lin C. M. Lee W. H. Wu A. T. Lin Y. K. Lin Y. F. (2015). Pterostilbene suppressed irradiation-resistant glioma stem cells by modulating GRP78/miR-205 axis. J. Nutr. Biochem. 26, 466–475. 10.1016/j.jnutbio.2014.11.015 25736407

[B33] Javed Z. Khan K. Rasheed A. Sadia H. Raza S. Salehi B. (2020). MicroRNAs and natural compounds mediated regulation of TGF signaling in prostate cancer. Front. Pharmacol. 11, 613464. 10.3389/fphar.2020.613464 33584291 PMC7873640

[B34] Jeon J. Olkhov-Mitsel E. Xie H. Yao C. Q. Zhao F. Jahangiri S. (2020). Temporal stability and prognostic biomarker potential of the prostate cancer urine miRNA transcriptome. J. Natl. Cancer Inst. 112, 247–255. 10.1093/jnci/djz112 31161221 PMC7073919

[B35] Jin Y. Zhou X. Yao X. Zhang Z. Cui M. Lin Y. (2020). MicroRNA-612 inhibits cervical cancer progression by targeting NOB1. J. Cell. Mol. Med. 24, 3149–3156. 10.1111/jcmm.14985 31970934 PMC7077537

[B36] Joshi T. Patel I. Kumar A. Donovan V. Levenson A. S. (2020). Grape powder supplementation attenuates prostate neoplasia associated with pten haploinsufficiency in mice fed high-fat diet. Mol. Nutr. Food Res. 64, e2000326. 10.1002/mnfr.202000326 32618118 PMC8103660

[B37] Kai L. Samuel S. K. Levenson A. S. (2010). Resveratrol enhances p53 acetylation and apoptosis in prostate cancer by inhibiting MTA1/NuRD complex. Int. J. Cancer 126, 1538–1548. 10.1002/ijc.24928 19810103

[B38] Kai L. Wang J. Ivanovic M. Chung Y. T. Laskin W. B. Schulze-Hoepfner F. (2011). Targeting prostate cancer angiogenesis through metastasis-associated protein 1 (MTA1). Prostate 71, 268–280. 10.1002/pros.21240 20717904

[B39] Kang C. H. Moon D. O. Choi Y. H. Choi I. W. Moon S. K. Kim W. J. (2011). Piceatannol enhances TRAIL-induced apoptosis in human leukemia THP-1 cells through Sp1- and ERK-dependent DR5 up-regulation. Toxicol. Vitro. 25, 605–612. 10.1016/j.tiv.2010.12.006 21167276

[B40] Kang N. J. Shin S. H. Lee H. J. Lee K. W. (2011). Polyphenols as small molecular inhibitors of signaling cascades in carcinogenesis. Pharmacol. Ther. 130, 310–324. 10.1016/j.pharmthera.2011.02.004 21356239

[B41] Kapetanovic I. M. Muzzio M. Huang Z. Thompson T. N. McCormick D. L. (2011). Pharmacokinetics, oral bioavailability, and metabolic profile of resveratrol and its dimethylether analog, pterostilbene, in rats. Cancer Chemother. Pharmacol. 68, 593–601. 10.1007/s00280-010-1525-4 21116625 PMC3090701

[B42] Kataria R. Khatkar A. (2019). Resveratrol in various pockets: A review. Curr. Top. Med. Chem. 19, 116–122. 10.2174/1568026619666190301173958 30834833

[B43] Khan J. Deb P. K. Priya S. Medina K. D. Devi R. Walode S. G. (2021). Dietary flavonoids: Cardioprotective potential with antioxidant effects and their pharmacokinetic, toxicological and therapeutic concerns. Molecules 26, 4021. 10.3390/molecules26134021 34209338 PMC8272101

[B44] Kolenda T. Guglas K. Kopczynska M. Sobocinska J. Teresiak A. Blizniak R. (2020). Good or not good: Role of miR-18a in cancer biology. Rep. Pract. Oncol. Radiother. 25, 808–819. 10.1016/j.rpor.2020.07.006 32884453 PMC7451592

[B45] Kumar A. Butt N. A. Levenson A. S. (2016). Natural epigenetic-modifying molecules in medical therapy. In Medical epigenetics, (United Kingdom: Elsevier), pp. pp.747–798.

[B46] Kumar A. Dhar S. Rimando A. M. Lage J. M. Lewin J. R. Zhang X. (2015). Epigenetic potential of resveratrol and analogs in preclinical models of prostate cancer. Ann. N. Y. Acad. Sci. 1348, 1–9. 10.1111/nyas.12817 26214308

[B47] Kumar A. Dholakia K. Sikorska G. Martinez L. A. Levenson A. S. (2019). MTA1-Dependent anticancer activity of Gnetin C in prostate cancer. Nutrients 11, E2096. 10.3390/nu11092096 31487842 PMC6770780

[B48] Kumar A. L. Levenson A. S. (2018). “Epigenetic mechanisms of resveratrol and its analogs in cancer prevention and treatment,” in Epigenetics of cancer prevention. Editors Bishayee A. Bhatia D. (United Kingdom: Elsevier), 169–186.

[B49] Latruffe N. Lancon A. Frazzi R. Aires V. Delmas D. Michaille J. J. (2015). Exploring new ways of regulation by resveratrol involving miRNAs, with emphasis on inflammation. Ann. N. Y. Acad. Sci. 1348, 97–106. 10.1111/nyas.12819 26190093

[B50] Levenson A. S. Kumar A. (2020). “Pterostilbene as a potent chemopreventive agent in cancer,” in Natural products for chemoprevention: single compounds and combinations. Editors Pezzuto J. M. Vang O. (United Kingdom: Springer Nature), 49–108.

[B51] Levenson A. S. Kumar A. Zhang X. (2014). MTA family of proteins in prostate cancer: biology, significance, and therapeutic opportunities. Cancer Metastasis Rev. 33, 929–942. 10.1007/s10555-014-9519-z 25332143

[B52] Levenson A. S. (2020). Metastasis-associated protein 1-mediated antitumor and anticancer activity of dietary stilbenes for prostate cancer chemoprevention and therapy. Semin. Cancer Biol. S1044-1579X (1020), 30045–30046. 10.1016/j.semcancer.2020.02.012 PMC7483334 32126261

[B53] Levenson A. S. (2021). Nitrients and phytonutrients as promising epigenetic nutraceuticals. In Medical epigenetics (United Kingdom: Elsevier). 741–816.

[B54] Li D. Zhang Y. Li Y. Wang X. Wang F. Du J. (2021). miR-149 suppresses the proliferation and metastasis of human gastric cancer cells by targeting FOXC1. Biomed. Res. Int. 2021, 1503403. 10.1155/2021/1503403 34957298 PMC8709748

[B55] Li J. Yen C. Liaw D. Podsypanina K. Bose S. Wang S. I. (1997). PTEN, a putative protein tyrosine phosphatase gene mutated in human brain, breast, and prostate cancer. Science 275, 1943–1947. 10.1126/science.275.5308.1943 9072974

[B56] Li K. Dias S. J. Rimando A. M. Dhar S. Mizuno C. S. Penman A. D. (2013). Pterostilbene acts through metastasis-associated protein 1 to inhibit tumor growth, progression and metastasis in prostate cancer. PLoS One 8, e57542. 10.1371/journal.pone.0057542 23469203 PMC3586048

[B57] Li X. Nie C. Tian B. Tan X. Han W. Wang J. (2019). miR-671-5p blocks the progression of human esophageal squamous cell carcinoma by suppressing FGFR2. Int. J. Biol. Sci. 15, 1892–1904. 10.7150/ijbs.32429 31523191 PMC6743296

[B58] Li X. Pan J. H. Song B. Xiong E. Q. Chen Z. W. Zhou Z. S. (2012). Suppression of CX43 expression by miR-20a in the progression of human prostate cancer. Cancer Biol. Ther. 13, 890–898. 10.4161/cbt.20841 22785209

[B59] Link A. Balaguer F. Goel A. (2010). Cancer chemoprevention by dietary polyphenols: Promising role for epigenetics. Biochem. Pharmacol. 80, 1771–1792. 10.1016/j.bcp.2010.06.036 20599773 PMC2974019

[B60] Liu W. H. Chang L. S. (2012). Suppression of Akt/Foxp3-mediated miR-183 expression blocks Sp1-mediated ADAM17 expression and TNFα-mediated NFκB activation in piceatannol-treated human leukemia U937 cells. Biochem. Pharmacol. 84, 670–680. 10.1016/j.bcp.2012.06.007 22705645

[B61] Liu Y. Lu L. L. Wen D. Liu D. L. Dong L. L. Gao D. M. (2020a). Correction to: MiR-612 regulates invadopodia of hepatocellular carcinoma by HADHA-mediated lipid reprogramming. J. Hematol. Oncol. 13, 44. 10.1186/s13045-020-00875-5 32366313 PMC7199356

[B62] Liu Y. Lu L. L. Wen D. Liu D. L. Dong L. L. Gao D. M. (2020b). MiR-612 regulates invadopodia of hepatocellular carcinoma by HADHA-mediated lipid reprogramming. J. Hematol. Oncol. 13, 12. 10.1186/s13045-019-0841-3 32033570 PMC7006096

[B63] Ma C. H. Zhang Y. X. Tang L. H. Yang X. J. Cui W. M. Han C. C. (2018). MicroRNA-1469, a p53-responsive microRNA promotes Genistein induced apoptosis by targeting Mcl1 in human laryngeal cancer cells. Biomed. Pharmacother. 106, 665–671. 10.1016/j.biopha.2018.07.005 29990856

[B64] Ma J. Wei H. Li X. Qu X. (2021). Hsa-miR-149-5p suppresses prostate carcinoma malignancy by suppressing RGS17. Cancer Manag. Res. 13, 2773–2783. 10.2147/CMAR.S281968 33790651 PMC8007479

[B65] Mak K. K. Wu A. T. Lee W. H. Chang T. C. Chiou J. F. Wang L. S. (2013). Pterostilbene, a bioactive component of blueberries, suppresses the generation of breast cancer stem cells within tumor microenvironment and metastasis *via* modulating NF-κB/microRNA 448 circuit. Mol. Nutr. Food Res. 57, 1123–1134. 10.1002/mnfr.201200549 23504987

[B66] Manavathi B. Kumar R. (2007). Metastasis tumor antigens, an emerging family of multifaceted master coregulators. J. Biol. Chem. 282, 1529–1533. 10.1074/jbc.R600029200 17142453

[B67] Matsushita M. Fujita K. Nonomura N. (2020). Influence of diet and nutrition on prostate cancer. Int. J. Mol. Sci. 21, 1447–1465. 10.3390/ijms21041447 PMC7073095 32093338

[B68] Miyata Y. Shida Y. Hakariya T. Sakai H. (2019). Anti-cancer effects of green tea polyphenols against prostate cancer. Molecules 24, E193. 10.3390/molecules24010193 30621039 PMC6337309

[B69] Moschini M. Carroll P. R. Eggener S. E. Epstein J. I. Graefen M. Montironi R. (2017). Low-risk prostate cancer: Identification, management, and outcomes. Eur. Urol. 72, 238–249. 10.1016/j.eururo.2017.03.009 28318726

[B70] Narayanan N. K. Kunimasa K. Yamori Y. Mori M. Mori H. Nakamura K. (2015). Antitumor activity of melinjo (Gnetum gnemon L.) seed extract in human and murine tumor models *in vitro* and in a colon-26 tumor-bearing mouse model *in vivo* . Cancer Med. 4, 1767–1780. 10.1002/cam4.520 26408414 PMC4674003

[B71] Olive V. Jiang I. He L. (2010). mir-17-92, a cluster of miRNAs in the midst of the cancer network. Int. J. Biochem. Cell Biol. 42, 1348–1354. 10.1016/j.biocel.2010.03.004 20227518 PMC3681296

[B72] Onder T. T. Gupta P. B. Mani S. A. Yang J. Lander E. S. Weinberg R. A. (2008). Loss of E-cadherin promotes metastasis *via* multiple downstream transcriptional pathways. Cancer Res. 68, 3645–3654. 10.1158/0008-5472.CAN-07-2938 18483246

[B73] Ostling P. Leivonen S. K. Aakula A. Kohonen P. Makela R. Hagman Z. (2011). Systematic analysis of microRNAs targeting the androgen receptor in prostate cancer cells. Cancer Res. 71, 1956–1967. 10.1158/0008-5472.CAN-10-2421 21343391

[B74] Overland M. R. Washington S. L. 3rd Carroll P. R. Cooperberg M. R. Herlemann A. (2019). Active surveillance for intermediate-risk prostate cancer: Yes, but for whom? Curr. Opin. Urol. 29, 605–611. 10.1097/MOU.0000000000000671 31436567

[B75] Pastor-Navarro B. Rubio-Briones J. Borque-Fernando A. Esteban L. M. Dominguez-Escrig J. L. Lopez-Guerrero J. A. (2021). Active surveillance in prostate cancer: Role of available biomarkers in daily practice. Int. J. Mol. Sci. 22, 6266. 10.3390/ijms22126266 34200878 PMC8230496

[B76] Peisch S. F. Van Blarigan E. L. Chan J. M. Stampfer M. J. Kenfield S. A. (2017). Prostate cancer progression and mortality: a review of diet and lifestyle factors. World J. Urol. 35, 867–874. 10.1007/s00345-016-1914-3 27518576 PMC5472048

[B77] Perl A. K. Wilgenbus P. Dahl U. Semb H. Christofori G. (1998). A causal role for E-cadherin in the transition from adenoma to carcinoma. Nature 392, 190–193. 10.1038/32433 9515965

[B78] Pesta M. Klecka J. Kulda V. Topolcan O. Hora M. Eret V. (2010). Importance of miR-20a expression in prostate cancer tissue. Anticancer Res. 30, 3579–3583. 20944140

[B79] Poliseno L. Salmena L. Riccardi L. Fornari A. Song M. S. Hobbs R. M. (2010). Identification of the miR-106b∼25 microRNA cluster as a proto-oncogenic PTEN-targeting intron that cooperates with its host gene MCM7 in transformation. Sci. Signal. 3, ra29. 10.1126/scisignal.2000594 20388916 PMC2982149

[B80] Qin W. Shi Y. Zhao B. Yao C. Jin L. Ma J. (2010). miR-24 regulates apoptosis by targeting the open reading frame (ORF) region of FAF1 in cancer cells. PLoS One 5, e9429. 10.1371/journal.pone.0009429 20195546 PMC2828487

[B81] Qin W. Zhang K. Clarke K. Weiland T. Sauter E. R. (2014). Methylation and miRNA effects of resveratrol on mammary tumors vs. normal tissue. Nutr. Cancer 66, 270–277. 10.1080/01635581.2014.868910 24447120

[B82] Quirico L. Orso F. (2020). The power of microRNAs as diagnostic and prognostic biomarkers in liquid biopsies. Cancer Drug resist. 3, 117–139. 10.20517/cdr.2019.103 35582611 PMC9090592

[B83] Rauf A. Imran M. Butt M. S. Nadeem M. Peters D. G. Mubarak M. S. (2018). Resveratrol as an anti-cancer agent: A review. Crit. Rev. Food Sci. Nutr. 58, 1428–1447. 10.1080/10408398.2016.1263597 28001084

[B84] Rimando A. M. Cuendet M. Desmarchelier C. Mehta R. G. Pezzuto J. M. Duke S. O. (2002). Cancer chemopreventive and antioxidant activities of pterostilbene, a naturally occurring analogue of resveratrol. J. Agric. Food Chem. 50, 3453–3457. 10.1021/jf0116855 12033810

[B85] Rimando A. M. Kalt W. Magee J. B. Dewey J. Ballington J. R. (2004). Resveratrol, pterostilbene, and piceatannol in vaccinium berries. J. Agric. Food Chem. 52, 4713–4719. 10.1021/jf040095e 15264904

[B86] Rimando A. M. Nagmani R. Feller D. R. Yokoyama W. (2005). Pterostilbene, a new agonist for the peroxisome proliferator-activated receptor alpha-isoform, lowers plasma lipoproteins and cholesterol in hypercholesterolemic hamsters. J. Agric. Food Chem. 53, 3403–3407. 10.1021/jf0580364 15853379

[B87] Riviere C. Pawlus A. D. Merillon J. M. (2012). Natural stilbenoids: distribution in the plant kingdom and chemotaxonomic interest in vitaceae. Nat. Prod. Rep. 29, 1317–1333. 10.1039/c2np20049j 23014926

[B88] Rudrapal M. Khairnar S. J. Khan J. Dukhyil A. B. Ansari M. A. Alomary M. N. (2022). Dietary polyphenols and their role in oxidative stress-induced human diseases: Insights into protective effects, antioxidant potentials and mechanism(s) of action. Front. Pharmacol. 13, 806470. 10.3389/fphar.2022.806470 35237163 PMC8882865

[B89] Rudrapal M. (2022). Phytoantioxidants and nanotherapeutics. Hoboken, NJ: Wiley.

[B90] Sapre N. Selth L. A. (2013). Circulating MicroRNAs as biomarkers of prostate cancer: The state of play. Prostate Cancer 2013, 539680. 10.1155/2013/539680 23577261 PMC3610368

[B91] Selth L. A. Townley S. Gillis J. L. Ochnik A. M. Murti K. Macfarlane R. J. (2012). Discovery of circulating microRNAs associated with human prostate cancer using a mouse model of disease. Int. J. Cancer 131, 652–661. 10.1002/ijc.26405 22052531

[B92] Shen T. Wang X. N. Lou H. X. (2009). Natural stilbenes: an overview. Nat. Prod. Rep. 26, 916–935. 10.1039/b905960a 19554241

[B93] Shenouda S. K. Alahari S. K. (2009). MicroRNA function in cancer: oncogene or a tumor suppressor? Cancer Metastasis Rev. 28, 369–378. 10.1007/s10555-009-9188-5 20012925

[B94] Sheth S. Jajoo S. Kaur T. Mukherjea D. Sheehan K. Rybak L. P. (2012). Resveratrol reduces prostate cancer growth and metastasis by inhibiting the Akt/MicroRNA-21 pathway. PLoS One 7, e51655. 10.1371/journal.pone.0051655 23272133 PMC3521661

[B95] Shukla S. Penta D. Mondal P. Meeran S. M. (2019). Epigenetics of breast cancer: Clinical status of epi-drugs and phytochemicals. Adv. Exp. Med. Biol. 1152, 293–310. 10.1007/978-3-030-20301-6_16 31456191

[B96] Song S. J. Poliseno L. Song M. S. Ala U. Webster K. Ng C. (2013). MicroRNA-antagonism regulates breast cancer stemness and metastasis *via* TET-family-dependent chromatin remodeling. Cell 154, 311–324. 10.1016/j.cell.2013.06.026 23830207 PMC3767157

[B97] Stoen M. J. Andersen S. Rakaee M. Pedersen M. I. Ingebriktsen L. M. Donnem T. (2021). Overexpression of miR-20a-5p in tumor epithelium is an independent negative prognostic indicator in prostate cancer-A multi-institutional study. Cancers (Basel) 13, 4096. 10.3390/cancers13164096 34439249 PMC8394585

[B98] Storniolo C. E. Quifer-Rada P. Lamuela-Raventos R. M. Moreno J. J. (2014). Piceid presents antiproliferative effects in intestinal epithelial Caco-2 cells, effects unrelated to resveratrol release. Food Funct. 5, 2137–2144. 10.1039/c4fo00305e 25007131

[B99] Su C. M. Lee W. H. Wu A. T. Lin Y. K. Wang L. S. Wu C. H. (2015). Pterostilbene inhibits triple-negative breast cancer metastasis *via* inducing microRNA-205 expression and negatively modulates epithelial-to-mesenchymal transition. J. Nutr. Biochem. 26, 675–685. 10.1016/j.jnutbio.2015.01.005 25792283

[B100] Su D. Cheng Y. Liu M. Liu D. Cui H. Zhang B. (2013). Comparision of piceid and resveratrol in antioxidation and antiproliferation activities *in vitro* . PLoS One 8, e54505. 10.1371/journal.pone.0054505 23342161 PMC3546968

[B101] Su L. David M. (2000). Distinct mechanisms of STAT phosphorylation *via* the interferon-alpha/beta receptor. Selective inhibition of STAT3 and STAT5 by piceatannol. J. Biol. Chem. 275, 12661–12666. 10.1074/jbc.275.17.12661 10777558

[B102] Taft R. J. Glazov E. A. Cloonan N. Simons C. Stephen S. Faulkner G. J. (2009). Tiny RNAs associated with transcription start sites in animals. Nat. Genet. 41, 572–578. 10.1038/ng.312 19377478

[B103] Tan Y. Y. Xu X. Y. Wang J. F. Zhang C. W. Zhang S. C. (2016). MiR-654-5p attenuates breast cancer progression by targeting EPSTI1. Am. J. Cancer Res. 6, 522–532. 27186421 PMC4859678

[B104] Taylor B. S. Schultz N. Hieronymus H. Gopalan A. Xiao Y. Carver B. S. (2010). Integrative genomic profiling of human prostate cancer. Cancer Cell 18, 11–22. 10.1016/j.ccr.2010.05.026 20579941 PMC3198787

[B105] Tili E. Michaille J. J. Adair B. Alder H. Limagne E. Taccioli C. (2010a). Resveratrol decreases the levels of miR-155 by upregulating miR-663, a microRNA targeting JunB and JunD. Carcinogenesis 31, 1561–1566. 10.1093/carcin/bgq143 20622002 PMC4647642

[B106] Tili E. Michaille J. J. Alder H. Volinia S. Delmas D. Latruffe N. (2010b). Resveratrol modulates the levels of microRNAs targeting genes encoding tumor-suppressors and effectors of TGFβ signaling pathway in SW480 cells. Biochem. Pharmacol. 80, 2057–2065. 10.1016/j.bcp.2010.07.003 20637737 PMC3918904

[B107] Tili E. Michaille J. J. (2011). Resveratrol, MicroRNAs, inflammation, and cancer. J. Nucleic Acids 2011, 102431. 10.4061/2011/102431 21845215 PMC3154569

[B108] Tili E. Michaille J. J. Wernicke D. Alder H. Costinean S. Volinia S. (2011). Mutator activity induced by microRNA-155 (miR-155) links inflammation and cancer. Proc. Natl. Acad. Sci. U. S. A. 108, 4908–4913. 10.1073/pnas.1101795108 21383199 PMC3064319

[B109] Urabe F. Kosaka N. Sawa Y. Ito K. Kimura T. Egawa S. (2020). The miR-1908/SRM regulatory axis contributes to extracellular vesicle secretion in prostate cancer. Cancer Sci. 111, 3258–3267. 10.1111/cas.14535 32558033 PMC7469824

[B110] Valeri N. Vannini I. Fanini F. Calore F. Adair B. Fabbri M. (2009). Epigenetics, miRNAs, and human cancer: a new chapter in human gene regulation. Mamm. Genome 20, 573–580. 10.1007/s00335-009-9206-5 19697081

[B111] Vanden Berghe W. (2012). Epigenetic impact of dietary polyphenols in cancer chemoprevention: lifelong remodeling of our epigenomes. Pharmacol. Res. 65, 565–576. 10.1016/j.phrs.2012.03.007 22465217

[B112] Venkatadri R. Muni T. Iyer A. K. Yakisich J. S. Azad N. (2016). Role of apoptosis-related miRNAs in resveratrol-induced breast cancer cell death. Cell Death Dis. 7, e2104. 10.1038/cddis.2016.6 26890143 PMC5399194

[B113] Ventura A. Jacks T. (2009). MicroRNAs and cancer: short RNAs go a long way. Cell 136, 586–591. 10.1016/j.cell.2009.02.005 19239879 PMC3910108

[B114] Vo N. T. Madlener S. Bago-Horvath Z. Herbacek I. Stark N. Gridling M. (2010). Pro- and anticarcinogenic mechanisms of piceatannol are activated dose dependently in MCF-7 breast cancer cells. Carcinogenesis 31, 2074–2081. 10.1093/carcin/bgp199 19696164

[B115] Waffo-Teguo P. Hawthorne M. E. Cuendet M. Merillon J. M. Kinghorn A. D. Pezzuto J. M. (2001). Potential cancer-chemopreventive activities of wine stilbenoids and flavans extracted from grape (Vitis vinifera) cell cultures. Nutr. Cancer 40, 173–179. 10.1207/S15327914NC402_14 11962253

[B116] Wang J. Ni J. Beretov J. Thompson J. Graham P. Li Y. (2020). Exosomal microRNAs as liquid biopsy biomarkers in prostate cancer. Crit. Rev. Oncol. Hematol. 145, 102860. 10.1016/j.critrevonc.2019.102860 31874447

[B117] Wang W. J. Yuan Y. Zhang D. Liu P. Liu F. (2021). miR-671-5p repressed progression of papillary thyroid carcinoma *via* TRIM14. Kaohsiung J. Med. Sci. 37, 983–990. 10.1002/kjm2.12424 34292652 PMC11896367

[B118] Wang Y. L. Shen Y. Xu J. P. Han K. Zhou Y. Yang S. (2017). Pterostilbene suppresses human endometrial cancer cells *in vitro* by down-regulating miR-663b. Acta Pharmacol. Sin. 38, 1394–1400. 10.1038/aps.2017.60 28552912 PMC5630673

[B119] Wang Y. Romigh T. He X. Orloff M. S. Silverman R. H. Heston W. D. (2010). Resveratrol regulates the PTEN/AKT pathway through androgen receptor-dependent and -independent mechanisms in prostate cancer cell lines. Hum. Mol. Genet. 19, 4319–4329. 10.1093/hmg/ddq354 20729295 PMC2957324

[B120] Wang Y. Zhang Q. Guo B. Feng J. Zhao D. (2020). miR-1231 is downregulated in prostate cancer with prognostic and functional implications. Oncol. Res. Treat. 43, 78–86. 10.1159/000504606 31822000

[B121] Xie G. P. Jiang R. (2015). Non-coding RNAs in castration-resistant prostate cancer. Zhonghua Nan Ke Xue 21, 1014–1019. 26738330

[B122] Xin C. Lu S. Li Y. Zhang Y. Tian J. Zhang S. (2019). miR-671-5p inhibits tumor proliferation by blocking cell cycle in osteosarcoma. DNA Cell Biol. 38, 996–1004. 10.1089/dna.2019.4870 31393166

[B123] Yu Y. Wang Z. Sun D. Zhou X. Wei X. Hou W. (2018). miR-671 promotes prostate cancer cell proliferation by targeting tumor suppressor SOX6. Eur. J. Pharmacol. 823, 65–71. 10.1016/j.ejphar.2018.01.016 29355560

[B124] Zhang H. Jia R. Wang C. Hu T. Wang F. (2014). Piceatannol promotes apoptosis *via* up-regulation of microRNA-129 expression in colorectal cancer cell lines. Biochem. Biophys. Res. Commun. 452, 775–781. 10.1016/j.bbrc.2014.08.150 25218158

[B125] Zhang T. Zhu X. Wu H. Jiang K. Zhao G. Shaukat A. (2019a). Targeting the ROS/PI3K/AKT/HIF-1α/HK2 axis of breast cancer cells: Combined administration of Polydatin and 2-Deoxy-d-glucose. J. Cell. Mol. Med. 23, 3711–3723. 10.1111/jcmm.14276 30920152 PMC6484306

[B126] Zhang W. Jiang H. Chen Y. Ren F. (2019b). Resveratrol chemosensitizes adriamycin-resistant breast cancer cells by modulating miR-122-5p. J. Cell. Biochem. 120, 16283–16292. 10.1002/jcb.28910 31155753

[B127] Zhao J. Li Q. Feng B. Wei D. Han Y. Li M. (2021). MicroRNA149 inhibits cancer cell malignant phenotype by regulating Akt1 in C42 CRPC cell line. Oncol. Rep. 46, 258. 10.3892/or.2021.8209 34698359 PMC8561672

[B128] Zhao Y. Cheng X. Wang G. Liao Y. Qing C. (2020). Linalool inhibits 22Rv1 prostate cancer cell proliferation and induces apoptosis. Oncol. Lett. 20, 289. 10.3892/ol.2020.12152 33029205 PMC7530887

[B129] Zhu J. Wang S. Zhang W. Qiu J. Shan Y. Yang D. (2015). Screening key microRNAs for castration-resistant prostate cancer based on miRNA/mRNA functional synergistic network. Oncotarget 6, 43819–43830. 10.18632/oncotarget.6102 26540468 PMC4791269

